# Identification and molecular characterization of *Wolbachia* strains in natural populations of *Aedes albopictus* in China

**DOI:** 10.1186/s13071-020-3899-4

**Published:** 2020-01-14

**Authors:** Yaping Hu, Zhiyong Xi, Xiaobo Liu, Jun Wang, Yuhong Guo, Dongsheng Ren, Haixia Wu, Xiaohua Wang, Bin Chen, Qiyong Liu

**Affiliations:** 1 National Institute for Communicable Disease Control and Prevention, CDC China, Beijing, China; 2 Nanjing Institute of Environmental Sciences, Ministry of Ecology and Environment of the People’s Republic of China, Nanjing, China; 3 Institute of Entomology and Molecular Biology, College of Life Sciences, Chongqing Normal University, Chongqing, China; 4 Key Laboratory of Tropical Disease Control of the Ministry of Education, Sun Yat-sen University–Michigan State University Joint Center of Vector Control for Tropical Diseases, Zhongshan School of Medicine, Sun Yat-sen University, Guangzhou, China; 5 Haikou Center for Disease Control and Prevention, Haikou, China

**Keywords:** *Aedes albopictus*, *Wolbachia*, Infection, MLST genes, Phylogenetic analysis, Dengue virus

## Abstract

**Background:**

*Aedes albopictus* is naturally infected with *Wolbachia* spp., maternally transmitted bacteria that influence the reproduction of hosts. However, little is known regarding the prevalence of infection, multiple infection status, and the relationship between *Wolbachia* density and dengue outbreaks in different regions. Here, we assessed *Wolbachia* infection in natural populations of *Ae. albopictus* in China and compared *Wolbachia* density between regions with similar climates, without dengue and with either imported or local dengue.

**Results:**

To explore the prevalence of *Wolbachia* infection, *Wolbachia* DNA was detected in mosquito samples *via* PCR amplification of the *16S* rRNA gene and the surface protein gene *wsp*. We found that 93.36% of *Ae*. *albopictus* in China were positive for *Wolbachia*. After sequencing *gatB*, *coxA*, *hcpA*, *ftsZ*, *fbpA* and *wsp* genes of *Wolbachia* strains, we identified a new sequence type (ST) of *w*AlbB (464/465). Phylogenetic analysis indicated that *w*AlbA and *w*AlbB strains formed a cluster with strains from other mosquitoes in a *wsp*-based maximum likelihood (ML) tree. However, in a ML tree based on multilocus sequence typing (MLST), *w*AlbB STs (464/465) did not form a cluster with *Wolbachia* strains from other mosquitoes. To better understand the association between *Wolbachia* spp. and dengue infection, the prevalence of *Wolbachia* in *Ae. albopictus* from different regions (containing local dengue cases, imported dengue cases and no dengue cases) was determined. We found that the prevalence of *Wolbachia* was lower in regions with only imported dengue cases.

**Conclusions:**

The natural prevalence of *Wolbachia* infections in China was much lower than in other countries or regions. The phylogenetic relationships among *Wolbachia* spp. isolated from field-collected *Ae. albopictus* reflected the presence of dominant and stable strains. However, *w*AlbB (464/465) and *Wolbachia* strains did not form a clade with *Wolbachia* strains from other mosquitoes. Moreover, lower densities of *Wolbachia* in regions with only imported dengue cases suggest a relationship between fluctuations in *Wolbachia* density in field-collected *Ae. albopictus* and the potential for dengue invasion into these regions.

## Background

Dengue is a rapidly spreading infectious disease transmitted between humans by mosquitoes of the genus *Aedes*. It is estimated that 400 million people are infected with dengue per year worldwide. To date, no effective vaccine or curative antiviral drug is available to prevent or treat dengue fever [1]. Thus, vector control has become the primary tool for dengue intervention. In China, *Aedes albopictus* is the primary dengue vector, and was responsible for the epidemic in 2014 resulting in approximately 47,000 infections. Use of insecticides is effective in controlling dengue, but is often prohibitively expensive, unsustainable and environmentally unfriendly. Other approaches require constant interventions that are expensive and difficult to implement in urban areas [2]. In recent years, the *Wolbachia-*based approach has been proposed as a new vector control strategy [3].

*Wolbachia* is a genus of Gram-negative bacteria that infect arthropods and filarial nematodes. It has been recently estimated that ~ 40% of arthropod species and ~ 28.1% of mosquitoes are infected with *Wolbachia* [4, 5]. These alpha-proteobacteria endosymbionts are transmitted vertically through host eggs and alter host biology in diverse ways, including reproductive manipulations such as feminization, parthenogenesis, male killing and sperm-egg incompatibility [6–8]. Furthermore, a large number of studies have shown that *Wolbachia* have an effect on the host’s olfactory sense, immunity and lifespan [9, 10]. After Hedges et al. [11] and Teixeira et al. [12] reported that *Wolbachia* can protect *Drosophila* flies from viral infections, a novel control strategy was proposed using *Wolbachia* to control or limit the spread of mosquito-transmitted diseases such as dengue and malaria. A *Wolbachia* strain from *Drosophila* could be transferred into *Aedes aegypti*; releasing this transinfected mosquito may result in invasion and spread of *Wolbachia* into wild mosquito populations [13]. Additionally, these strains also interfere with the host’s reproduction, inhibit viral replication and reduce adult lifespan [14].

*w*Mel-transinfected *Ae. aegypti* populations have already been established and successfully released in Australia [3, 15]. Subsequently, other countries and regions in which *Ae. aegypti* is the main vector of dengue, such as Vietnam, Brazil, Colombia and Indonesia, have also started to release *w*Mel-infected mosquitoes [16, 17]. In different parts of China, especially the south (e.g. Guangdong), *Ae. albopictus* is the major vector of dengue. Thus, studies are currently underway to apply a *Wolbachia* strain, *w*Pip, from a *Culex* mosquito species to control *Ae. albopictus*. Although the theory and technology are already established, the prevalence and characteristics of *Wolbachia* in natural *Ae. albopictus* populations are poorly understood.

*Aedes albopictus* carries *Wolbachia* superinfections with two strains, *w*AlbA and *w*AlbB. In a given region *Ae. albopictus* harbors only single *w*AlbA infections, and field-collected mosquitoes with single *w*AlbB infections were identified in Changsha, Chenzhou and Wuhan, as has been previously reported in Guangzhou [18]. Studies of natural *Wolbachia* infections of *Ae. albopictus* in China have been much less conclusive and were mainly based on the *wsp* gene. In addition, multilocus sequence typing (MLST), a robust classification system that accomplishes strain typing based on variation in five conserved housekeeping genes (*ftsZ*, *gatB*, *coxA*, *hcpA* and *fbpA*), was applied in mosquitoes singly infected with supergroup A or B *Wolbachia* [19]. No studies have applied MLST to assess co-infection with supergroups A and B *Wolbachia* in *Ae. albopictus*. In previous studies, quantification of *Wolbachia* in mosquitoes aimed to examine the direct association between *Wolbachia* and virus *in vivo*, and several studies were carried out to understand virus-*Wolbachia* relationships in natural mosquito populations [20, 21].

The present study aimed to determine the natural prevalence of *Wolbachia* infections and to investigate differences in *Wolbachia* infection among five different climatic regions. MLST and *wsp* analyses were applied to characterize *Wolbachia* strains and estimate the phylogenetic relationships between *Wolbachia* strains in field-collected *Ae. albopictus* from China. Our findings illuminate the characteristics and prevalence of *Wolbachia* in natural populations of *Ae. albopictus* in China.

## Methods

### Mosquito sampling

**Fig. 1:**
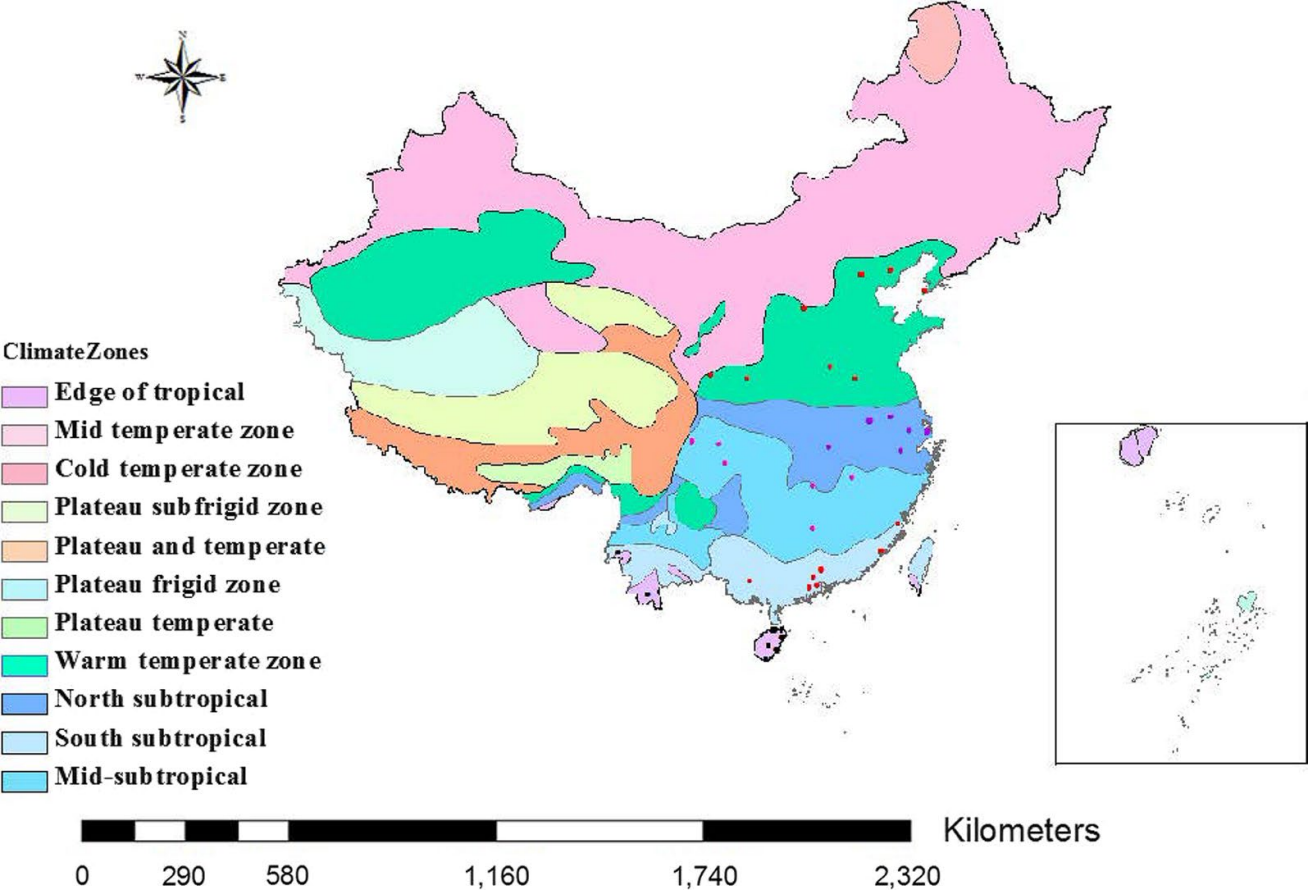
Distribution of sampling sites for *Ae. albopictus*. Black, red, pink, purple, and brown dots are the sample sites at the Edge of tropical, South subtropical, Mid-subtropical, North subtropical and Warm temperate zones, respectively

According to the geographical distribution and climatic characteristics of *Ae. albopictus* in China, we selected 6–8 sites in each of five climate zones of *Ae. albopictus* distribution. Samples were collected at each site according to a five-point method. In this study, a total of 704 adult *Ae. albopictus* (190 males and 514 females) were collected from 34 districts between June and October 2014 (Table 1). For analysis of prevalence, sampling locations were placed into five climate groups as defined in the Chinese Climatic Regions, based on the following climate classifications: Edge of tropical; South subtropical; Mid-subtropical; North subtropical; and Warm temperate zone (Fig. 1) [22]. BG traps, human baited net traps and manual aspirators were used to catch adult mosquitoes. Pipettes and dippers were used for capturing larvae or pupae from different containers at each site. The same operation was repeated at least five times in each location to reduce sampling error. Sampling staff were well protected whilst catching adults to avoid mosquito bites. The collected larvae and pupae were reared to adults and supplemented with yeast extract. The adults collected in the field were examined morphologically to confirm whether they were *Ae. albopictus* [23]. Samples were stored at − 80 °C in individual tubes containing 95% ethanol until DNA extraction.

**Table 1:** Sample information

Climate zone	District	Coordinates	No. of samples	♀	♂
Edge of tropical	Wenchang	19.57°N, 110.80°E	33	25	8
	Wanning	18.81°N, 110.39°E	21	16	5
	Haikou	20.02°N, 110.20°E	33	21	12
	Qiongzhong	19.04°N, 109.83°E	18	10	8
	Sanya	18.25°N, 109.51°E	37	22	15
	Jinghong	22.01°N, 100.77°E	34	23	11
	Dehong	24.43°N, 98.59°E	21	19	2
South subtropical	Nanning	22.82°N, 108.36°E	25	17	8
	Foshan	23.02°N, 113.11°E	12	10	2
	Guangzhou	23.41°N, 113.23°E	33	19	14
	Jiangmen	22.50°N, 113.40°E	5	2	3
	Zhongshan	22.40°N, 112.72°E	20	12	8
	Fuzhou	26.08°N, 119.30°E	24	18	6
	Xiamen	24.59°N, 118.10°E	24	15	9
Mid-subtropical	Changsha	28.21°N, 112.99°E	25	11	14
	Chenzhou	25.77°N, 113.01°E	18	13	5
	Nanchang	28.68°N, 115.86°E	22	16	6
	Chengdu	30.66°N, 104.07°E	18	14	4
	Nanchong	30.49°N, 106.04°E	2	1	1
	Chongqing	29.57°N, 106.55°E	24	24	24
North subtropical	Hefei	31.82°N, 117.23°E	9	8	1
	Nanjing	32.05°N, 118.79°E	27	20	7
	Shanghai	31.23°N, 121.48°E	31	24	7
	Wuhan	30.35°N, 114.17°E	6	2	4
	Wuxi	31.34°N, 120.18°E	3	3	0
	Hangzhou	30.18°N, 119.5°E	26	18	8
Warm temperate zone	Beijing	39.77°N, 116.66°E	30	24	6
	Shangqiu	34.17°N, 116.20°E	13	13	0
	Taiyuan	37.98°N, 112.32°E	31	31	0
	Xian	34.17°N, 108.21°E	18	18	0
	Tangshan	39.96°N, 118.81°E	3	2	1
	Kaifeng	34.80°N, 114.27°E	15	12	3
	Tianshui	34.71°N, 105.47°E	22	20	2
	Dalian	38.94°N, 121.40°E	21	15	6

### DNA extraction and prevalence of *Wolbachia* infection

To assess the prevalence of *Wolbachia* infection, 2–37 *Ae. albopictus* were used from each population to extract total DNA. After drying the *Ae. albopictus* for several minutes, they were washed three times in ddH_2_O. DNA was then individually extracted using a DNAeasy Tissue Kit (Qiagen, Valencia, CA, USA). Two *16S* rDNA primers and four *wsp*-specific primers, WAF/WAR and WBF/WBR, were used to detect *Wolbachia* DNA by polymerase chain reaction (PCR) using the DNA of a single mosquito as a template [24, 25]. The *28S* rRNA gene was used to assess the quality of DNA extraction and the *cox*1 mitochondrial gene was sequenced to exclude mosquitoes that were not *Ae. albopictus*. The full-length *cox*1 gene was amplified using four primers, *cox*1F/*cox*1R and *cox*1f/*cox*1r (Table 2). PCR reactions were performed in a final volume of 25 μl containing 2 μl of DNA, 11 μl of ddH_2_O, 1 μM of each primer and 10 μl of SuperMix. The temperature was cycled at 94 °C for 2 min, followed by 37 cycles of 94 °C for 30 s, 55 °C for 45 s and 72 °C for 1 min, and then a final extension step at 72 °C for 10 min. DNA extracted from *Wolbachia*-infected *Ae. albopictus* was used as a positive control and ddH_2_O was used as a negative control. PCR products were run on 1% agarose gels and the *cox*1 PCR products were sequenced directly.

**Table 2:** Primers for amplification and sequencing

Gene	Primer	Sequence (5′–3′)	Annealing T (°C)
*16S* rDNA	16SF	CGGGGGAAAAATTTATTGCT	55
	16SR	AGCTGTAATACAGAAAGTAAA	
wAlbA-*wsp*	WAF	CCAGCAGATACTATTGCG	55
	WAR	AAAAATTAAACGCTACTCCA	
wAlbB-*wsp*	WBF	AAGGAACCGAAGTTCATG	55
	WBR	AAAAATTAAACGCTACTCCA	
*wsp*	81	TGGTCCAATAAGTGATGAAGAAAC	53
	691	AAAAATTAAACGCTACTCCA	
*FtsZ*	ftsZ-F	TACTGACTGTTGGAGTTGTAACTAAGCCGT	58
	ftsZ-R	TGCCAGTTGCAAGAACAGAAACTCTAACTC	
*28S* rRNA	28F	TACCGTGAGGGAAAGTTGAAA	55
	28R	AGACTCCTTGGTCCGTGTTT	
*cox*1	cox1F	TTTACAATTTATCGCCTAAACTTC	55
	cox1R	CATTGCACTAATCTGCCATA	
	cox1f	GGGGGAGACCCTATTTTATA	55
	cox1r	TAAACTTCAGGGTGACCAAAAAATCA	
wAlbAq-*wsp*	qAF	GGGTTGATGTTGAAGGAG	55
	qAR	CACCAGCTTTTACTTGACC	
wAlbBq-*wsp*	qBF	ACGTTGGTGGTGCAACATTTG	58
	qBR	TAACGAGCACCAGCATAAAGC	
RPS	RPS6-F	CGTCGTCAGGAACGTATTCG	55
	RPS6-R	TCTTGGCAGCCTTGACAGC	

*Note*: Primers *cox*1f/*cox*1r were used for sequencing

*Abbreviation*: T, temperature

### Cloning and sequencing of *wsp* and MLST genes

The WSP loci were amplified with *wsp* (*Wolbachia* surface protein gene) primers to confirm multiple infections. PCR reactions were performed in a final volume of 25 μl containing 2 μl of DNA, 11 μl of ddH_2_O, 1 μM of each primer and 10 μl of SuperMix. The temperature was cycled at 94 °C for 2 min, followed by 37 cycles of 94 °C for 30 s, 53 °C for 45 s and 72 °C for 1 min, and then a final extension step at 72 °C for 10 min.

The five MLST loci were amplified according to previously published protocols (http://pubmlst.org/Wolbachia/). PCR reactions were performed in a final volume of 25 μl containing 2 μl of DNA, 11 μl of ddH_2_O, 1 μM of each primer and 10 μl of SuperMix. The temperature was cycled at 94 °C for 2 min, followed by 37 cycles of 94 °C for 30 s, T_m_ (T_m_ values for each primer pair are shown in Table 2) for 45 s and 72 °C for 90 s, and then a final extension step at 72 °C for 10 min. For co-infected samples, the *coxA* and *ftsZ* genes were amplified using primers *coxA*_F1 (5′-TTG GRG CRA TYA ACT TTA TAG-3′) and *coxA*_R1 (5′-CT AAA GAC TTT KAC RCC AGT-3′), and *fts*Z-F (5′-TAC TGA CTG TTG GAG TTG TAA CTA AGC CGT-3′) and *fts*Z-R (5′-TGC CAG TTG CAA GAA CAG AAA CTC TAA CTC-3′), respectively. For the fragment of *coxA*, primers for B-specific MLST protocols for AB infections were not used in our study, and *ftsZ* fragments were not long enough to be amplified by A-specific and B-specific primers. Fragments of *coxA*, *ftsZ* and *wsp* with the expected sizes were excised from the gel and purified using the Pure Yield™ Plasmid Miniprep System (Promega, Madison, USA). The purified DNA was ligated into pEASY-T5 Zero Cloning vector (Trans) and then transferred to *Trans*1-T1 phage resistant chemically competent cells (Trans). Putative clones of expected fragments were submitted for DNA sequencing. For all three kinds of fragments, at least eight clones were sequenced for each mosquito using both M13 forward and reverse primers, with three individuals being analyzed for each geographical population.

### Nucleotide sequence accession numbers

All newly generated sequences for *wsp*, *cox*1, *gatB*, *coxA*, *hcpA*, *ftsZ*, *fbpA* genes were deposited in the GenBank database under accession numbers KU738304-KU738385, KU738386-KU738431, MK809569-MK809640, MK809709-MK809776, MK809845-MK809912, MK809777-MK809844, MK809641-MK809708, respectively. According to the MLST protocol, the sequences of *gatB*, *coxA*, *hcpA*, *ftsZ*, *fbpA* and *wsp* were submitted to the PubMLST database for sequence typing, generating a MLST allelic profile and a WSP hypervariable region (HVR) profile. Strain and host information were deposited in the MLST database.

### Sequence typing and phylogenetic analyses

For *Wolbachia*-specific *wsp* gene sequence analysis, several reported sequences with similarities of > 97% were obtained from GenBank for comparisons. The *wsp* sequence of *Brugia malayi* was selected as the outgroup. We also analyzed co-infection with different *Wolbachia* species. Furthermore, a reference list of *Wolbachia* isolates was constructed by searching the MLST database, which was selected for having a complete set of MLST and HVR profiles. A total of 40 of known STs were from supergroup A, supergroup B, supergroup D and supergroup F *Wolbachia*, and supergroup D (Table 3) and supergroup F *Wolbachia* were selected as outgroups. Allele sequences were downloaded from the MLST database and these *Wolbachia* sequences were manually edited with Chromas2.4 by DNAMAN and their translated amino acid sequences were aligned using MUSCLE in MEGA6.0. Then, the concatenated data set of the five MLST genes was subjected to a phylogenetic analysis using MEGA 6.0. The *wsp* sequences were also subjected to a phylogenetic analysis using MEGA 6.0 using supergroup D and F *Wolbachia* strains as outgroups (for consistency with the MLST-based analysis). Maximum likelihood (ML) methods in MEGA 6.0 were used to analyze phylogenetic relationships. To select the optimal evolutionary model by critically evaluating the selected parameters, Find Best-Fit Substitution Model was conducted in MEGA 6.0 [26]. For the NCBI-*wsp* sequences, the concatenated dataset and the *wsp* sequences, the submodels T92 (Tamura 3-parameter), GTR+I+G and T92 (Tamura 3-parameter)+G were selected, respectively. The ML trees were constructed with 1000 bootstrap replicates.

**Table 3:** MLST allelic and WSP profiles of *Wolbachia* subjected for phylogenetic analyses

ID	Supergroup	Host species	ST	*gatB*	*coxA*	*hcpA*	*ftsZ*	*fbpA*	wsp	*HVR1*	*HVR2*	*HVR3*	*HVR4*
1	A	*Drosophila melanogaster*	1	1	1	1	1	1	31	1	12	21	24
12	A	*Aedes albopictus*	2	3	2	2	10	3	1	1	1	1	1
496	A	*Aedes bromeliae*	304	182	160	187	148	232					
114	A	*Notoncus* sp.	53	46	42	23	6	17	49	9	9	12	9
120	A	*Camponotus leonardi*	57	49	44	53	42	49	52	41	42	45	42
167	A	*Agelenopsis aperta*	67	35	35	22	33	39	43	31	32	35	34
294	A	*Asobara japonica*	370	87	111	103	70	186	530	188	213	15	25
399	A	*Apanteles chilonis*	260	172	150	7	137	8	592	209	15	17	14
1682	A	*Syrphophilus asperatus*	433	234	84	257	200	120	689	11	9	267	302
56	A	*Rhagoletis cerasi*	13	1	1	1	3	1	23	1	12	21	11
2	A	*Solenopsis invicta*	29	19	20	22	17	20	28	21	21	25	21
61	A	*Rhagoletis cerasi*	159	53	84	85	70	79	113	67	77	12	9
68	A	*Agelenopsis aperta*	65	32	33	38	30	37	38	28	29	33	32
88	A	*Drosophila testacea*	99	10	72	11	14	11	13	1	11	21	11
107	A	*Wasmannia* Peru	47	43	20	46	38	46	28	21	21	25	21
96	A	*Aganaspis alujai*	164	54	52	62	82	62	75	11	9	15	25
129	A	*Dorymyrmex elegans*	63	19	21	55	46	53	51	42	43	47	25
325	A	*Ephestia kuehniella*	92	54	59	68	3	67	83	51	55	15	57
413	A	*Chelonus munakatae*	19	7	6	7	3	8	599	2	191	192	248
29	B	*Culex pipiens*	9	4	3	3	22	4	10	10	8	10	8
499	B	*Mansonia africana*	305	9	38	189	36	4					
19	B	*Chelymorpha alternans*	7	9	14	15	12	14	8	7	7	8	7
22	B	*Acraea encedon*	3	9	11	12	11	12	2	2	2	2	2
27	B	*Drosophila simulans*	16	5	4	4	4	5	15	10	8	11	13
34	B	*Nasonia vitripennis*	26	9	8	9	7	9	25	18	16	23	16
99	B	*Horaga onyx*	39	12	14	13	2	41	65	34	36	3	23
118	B	*Pheidole sciophila*	56	48	43	52	41	6	60	40	41	43	41
408	B	*Apanteles chilonis*	271	9	150	7	142	4	593	18	79	237	16
269	B	*Diaphorina Diaphorina citri*	175	109	86	88	126	27	160	2	17	3	23
39	B	*Lycaeides idas*	36	9	36	40	7	9	61	18	16	23	16
73	B	*Lycaeides melissa*	162	108	73	40	80	9	294	125	141	127	102
70	B	*Rhagoletis cerasi*	160	101	85	40	22	4	116	69	17	3	23
40	B	*Hypolimnas bolina*	125	4	14	40	73	4	10	10	8	10	8
87	B	*Drosophila innubila*	98	79	71	88	69	27	82	2	35	98	23
97	B	*Anthene emolus*	37	9	9	6	8	10	63	19	17	24	33
200	B	*Eurema mandarina*	40	38	38	29	35	42	64	35	35	38	44
311	B	*Sogatella furcifera*	213	106	11	13	105	162	463	2	191	192	22
315	B	*Macrosteles fascifrons*	217	135	120	141	108	197	536	191	220	23	16
37	D	*Brugia malayi*	35	28	29	33	26	30	34	24	24	27	26
36	F	*Cimex lectularius*	8	26	27	31	24	28	7	6	6	7	6

### *w*AlbA and *w*AlbB *Wolbachia* strain quantitation

Twenty-eight mosquitoes from regions with local dengue cases (Guangzhou and Jinghong), with only imported cases (Xiamen and Haikou) and without dengue cases (Wenchang and Fuzhou) were amplified individually by quantitative PCR using strain-specific primers qAF/qAR [27] and qBF/qBR (Table 2) to examine the relationship between *Wolbachia* density in field-collected *Ae. albopictus* and the presence of dengue virus. The Bio-Rad CFX96 Real-Time PCR Detection System (Hercules, USA) and GoTaq® qPCR Master Mix (Promega) were used in our study. PCR reactions were performed in a final volume of 20 µl containing 10 µl of GoTaq® qPCR Master Mix, 0.5 µM of each primer, 2 µl of template DNA and 7 µl of RNase-free water. Reactions were mixed with an electronic pipette. The thermal cycling conditions were: 10 min at 95 °C, followed by 50 cycles of 94 °C for 15 s, primer T_m_ (*w*AlbA 55 °C, *w*AlbB 58 °C and RPS6 55 °C) for 30 s, 72 °C for 30 s, and finally 72 °C (read temperature) for 15 s. The melting curve was constructed between 49 °C and 63 °C. We used a serial dilution of *pEASY*®-T5 Zero Cloning vectors containing one copy each of RPS6 [28], *w*AlbAq-*wsp* and *w*AlbBq-*wsp* gene fragments, and used their primers set up in each PCR to plot standard curves, in case any binding efficiency difference appeared. Every mosquito DNA template was quantified three times for each of the RPS6, *w*AlbAq-*wsp* and *w*AlbBq-*wsp* genes. Assuming that each gene was present in a single copy per haploid genome, the ratio between *wsp* and RPS6 provided the number of *Wolbachia* genomes relative to the number of *Aedes* genomes [29].

### Statistical analysis

To compare the densities of the two *Wolbachia* strains in field mosquitoes in five regions (with different adult sizes), data were normalized to the expression of the host *rps6* gene. Analyses were carried out using SPSS Statistics (17.0). Chi-square tests were performed to compare the prevalence of *Wolbachia* infections and one-way analysis of variance (ANOVA) was performed to compare densities of *Wolbachia* from different regions for normally distributed data using SPSS Statistics (17.0). Differences were considered statistically significant when *P* < 0.05. For better presentation of results, locA and locB were used to denote the densities of supergroup A and supergroup B, respectively, from regions with local dengue cases; impA and impB were used to denote the densities of supergroup A and supergroup B, respectively, from regions with only imported dengue cases; and noA and noB were used to denote the densities of supergroup A and supergroup B, respectively, from regions with no dengue cases.

## Results

### Prevalence of *Wolbachia* infections

A total of 693 adult *Ae. albopictus* were obtained from five different climatic regions in China and were examined for *Wolbachia* infection status. Of these, 93.36% (647/693) were PCR-positive for *Wolbachia* using *wsp* and *16S* rDNA primers [30]. The quality of extracted DNA was good, and the samples were all identified as *Ae. albopictus* [31]. Specific primers for *w*AlbA and *w*AlbB, derived from the rapidly evolving *wsp* outer-surface protein gene of *Wolbachia*, were used to screen for these bacteria in *Ae. albopictus* mosquitoes. The PCR results showed that 83.26% (577/693) of the mosquitoes sampled were infected with supergroup A and 91.05% (631/693) were infected with supergroup B *Wolbachia* strains. The prevalence of co-infection was 80.95% (561/693). Individuals singly infected with supergroup A and supergroup B *Wolbachia* represented 2.31% (16/693) and 10.10% (70/693) of all mosquitoes, respectively. We also found 46 uninfected individuals (Table 4).

**Table 4:** Infection status of *Wolbachia* based on PCR results of field-collected *Ae*. *albopictus* adults

Climate region	Total	No. of infected (%)			
		Single A	Single B	A and B	W+
Edge of tropical	186	11 (5.91)	26 (13.98)	135 (72.58)	172 (92.47)
South subtropical	143	1 (0.70)	15 (10.49)	117 (81.82)	133 (93.01)
Mid-subtropical	109	1 (0.92)	13 (11.93)	80 (73.39)	94 (86.24)
North subtropical	102	2 (1.96)	12 (11.76)	85 (83.33)	99 (97.06)
Warm temperate zone	153	1 (0.65)	4 (2.61)	144 (94.12)	149 (97.39)
Total	693	16 (2.31)	70 (10.10)	561 (80.95)	647 (93.36)

*Note*: W+ represents the positive rate of *Wolbachia* in *Ae*. *albopictus*

**Fig. 2:**
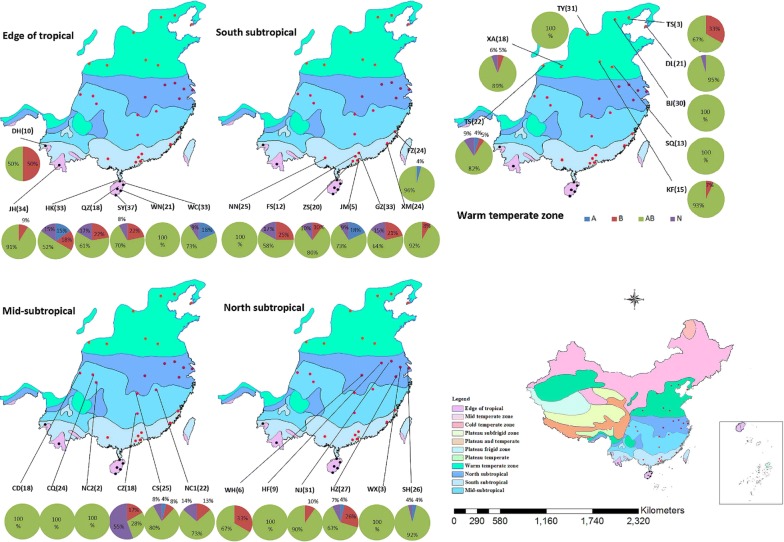
Infection rates for different sites in the Edge of tropical, South subtropical, Mid-subtropical, North subtropical and Warm temperate zones. Black, red, pink, purple and brown dots are the sample sites at the edge of Tropical, South subtropical, Mid-subtropical, North subtropical and Warm temperate zones, respectively. Blue square A, rate of single-infected with *w*AlbA mosquitoes; brown square B, rate of single-infected with *w*AlbB mosquitoes; green square AB, rate of co-infected with *w*AlbA and *w*AlbB mosquitoes; purple square N, rate of *Wolbachia*-negative mosquitoes

**Fig. 3:**
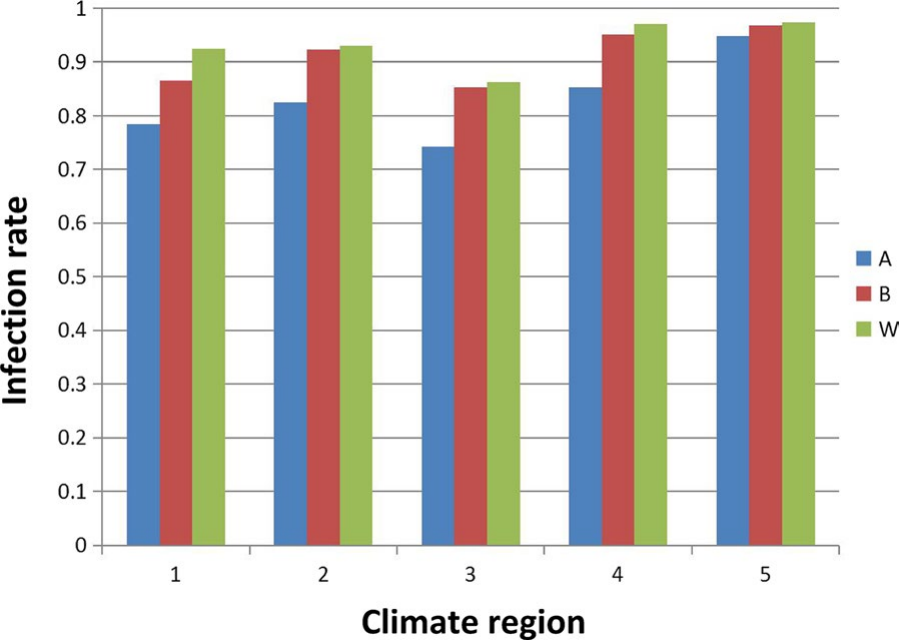
*Wolbachia* infection rates in *Ae. albopictus* of the five climate regions in China: 1, Edge of tropical; 2, South subtropical; 3, Mid-subtropical; 4, North subtropical; 5, Warm temperate zone. Blue bars (A), *w*AlbA infection rate in *Ae. albopictus*; brown bars (B), *w*AlbB infection rate in *Ae. albopictus*; green bars (W), rate of co-infection with *w*AlbA and *w*AlbB in *Ae. albopictus*

The natural prevalence of *Wolbachia* infection in 34 different locations of the five climatic regions is presented in Fig. 1. Chi-square tests of *Wolbachia* prevalence among the five different climate regions (Fig. 2) revealed a significant difference (*χ*^2^ = 15.438, *df* = 4, *P* = 0.004). Similarly, the prevalence of supergroup A and B *Wolbachia* differed significantly among the five climate regions (*χ*^2^ = 24.199, *df* = 4, *P* < 0.0001 and *χ*^2^ = 17.390, *df* = 4, *P* = 0.0020, respectively). Further analysis showed that the prevalence of *Wolbachia* infection was significantly different in four regions: Edge of tropical *vs* Warm temperate zone (*χ*^2^ = 4.029, *df* = 1, *P* = 0.045); Mid-subtropical *vs* North subtropical (*χ*^2^ = 7.906, *df* = 1, *P* = 0.005); and Mid-subtropical *vs* Warm temperate zone (*χ*^2^ = 11.759, *df* = 1, *P* = 0.001). However, the prevalence of *Wolbachia* infection in the South subtropical region did not show any significant differences compared with any of the other four regions. For the prevalence of supergroup A *Wolbachia*, significant differences were detected for five regions: Edge of tropical *vs* Warm temperate zone (*χ*^2^ = 18.298, *df* = 1, *P* < 0.0001); South subtropical *vs* Warm temperate zone (*χ*^2^ = 11.204, *df* = 1, *P* = 0.001); Mid-subtropical *vs* North subtropical (*χ*^2^ = 3.917, *df* = 1, *P* = 0.048); Mid-subtropical *vs* Warm temperate zone (*χ*^2^ = 22.480, *df* = 1, *P* < 0.0001); and North subtropical *vs* Warm temperate zone (*χ*^2^ = 6.698, *df* = 1, *P* = 0.010). For supergroup B *Wolbachia*, significant differences were observed in four regions: Edge of tropical *vs* North subtropical (*χ*^2^ = 5.147, *df* = 1, *P* = 0.023); Edge of tropical *vs* Warm temperate zone (*χ*^2^ = 10.770, *df* = 1, *P* = 0.001); Mid-subtropical *vs* North subtropical (*χ*^2^ = 5.620, *df* = 1, *P* = 0.018); and Mid-subtropical *vs* Warm temperate zone (*χ*^2^ = 11.242, *df* = 1, *P* = 0.001). Similar to the overall prevalence of *Wolbachia* infections, the south subtropical region did not show any substantial difference compared with any of the other four regions (Figs. 2, 3).

### Nucleotide sequence analysis of *Wolbachia* from *Ae. albopictus*

**Fig. 4:**
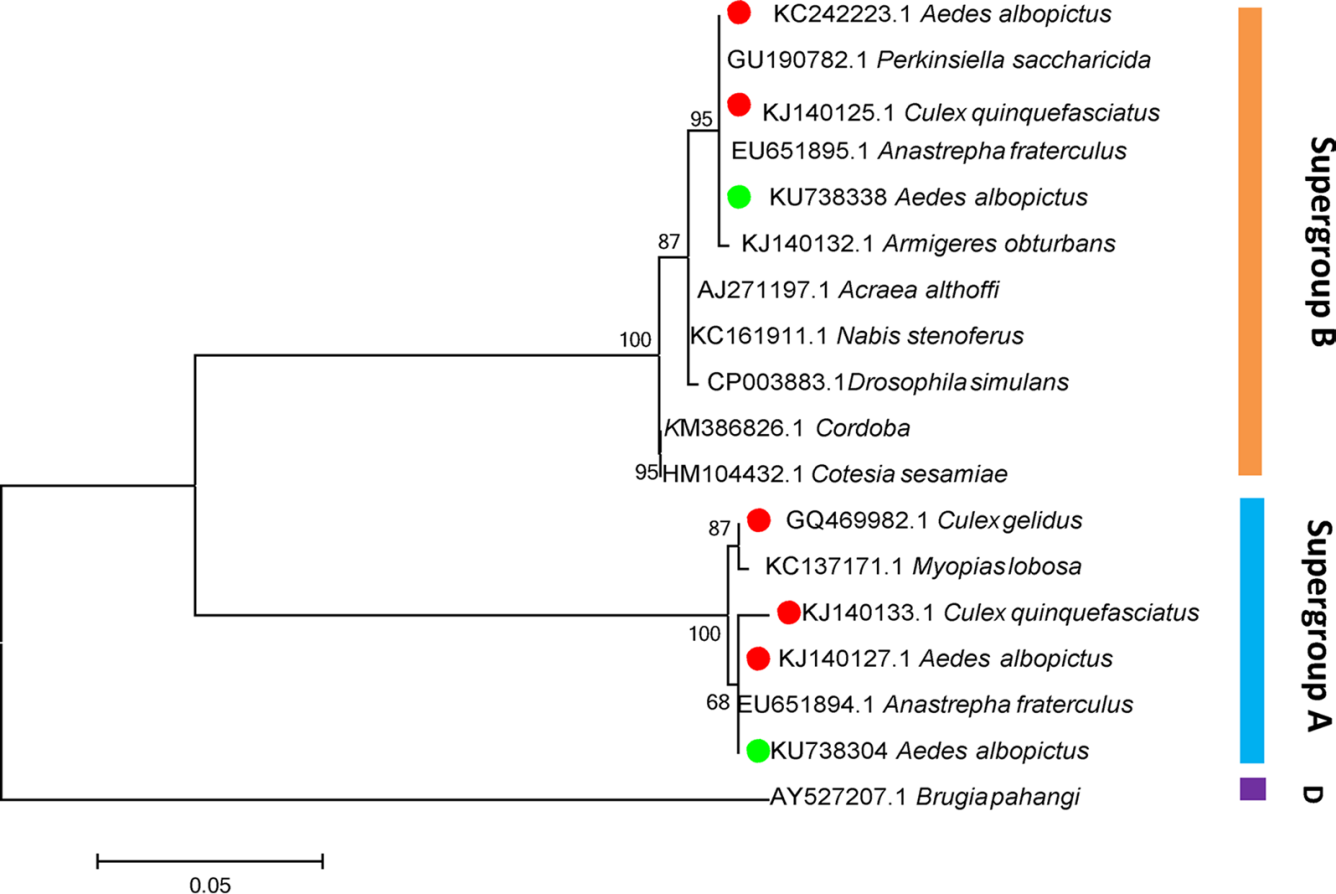
Maximum likelihood phylogenetic tree based on *wsp* gene sequences for *Wolbachia* from different hosts from GenBank. Red dots indicate reported *Wolbachia* strains of mosquitoes; green dots indicate *Wolbachia* strains of *Ae. albopictus* sampled in the present study

DNA sequencing analysis indicated that *Ae. albopictus* from different locations in China harbored two different *Wolbachia* strains: *w*AlbA and *w*AlbB (Fig. 4). The WSP profiles of *w*AlbA and *w*AlbB for *wsp*, HVR1, HVR2, HVR3 and HVR4 were 1, 1, 1, 1 and 1, and 169, 10, 82, 10 and 84, respectively, suggesting that these two *Wolbachia* strains were very stable.

**Fig. 5:**
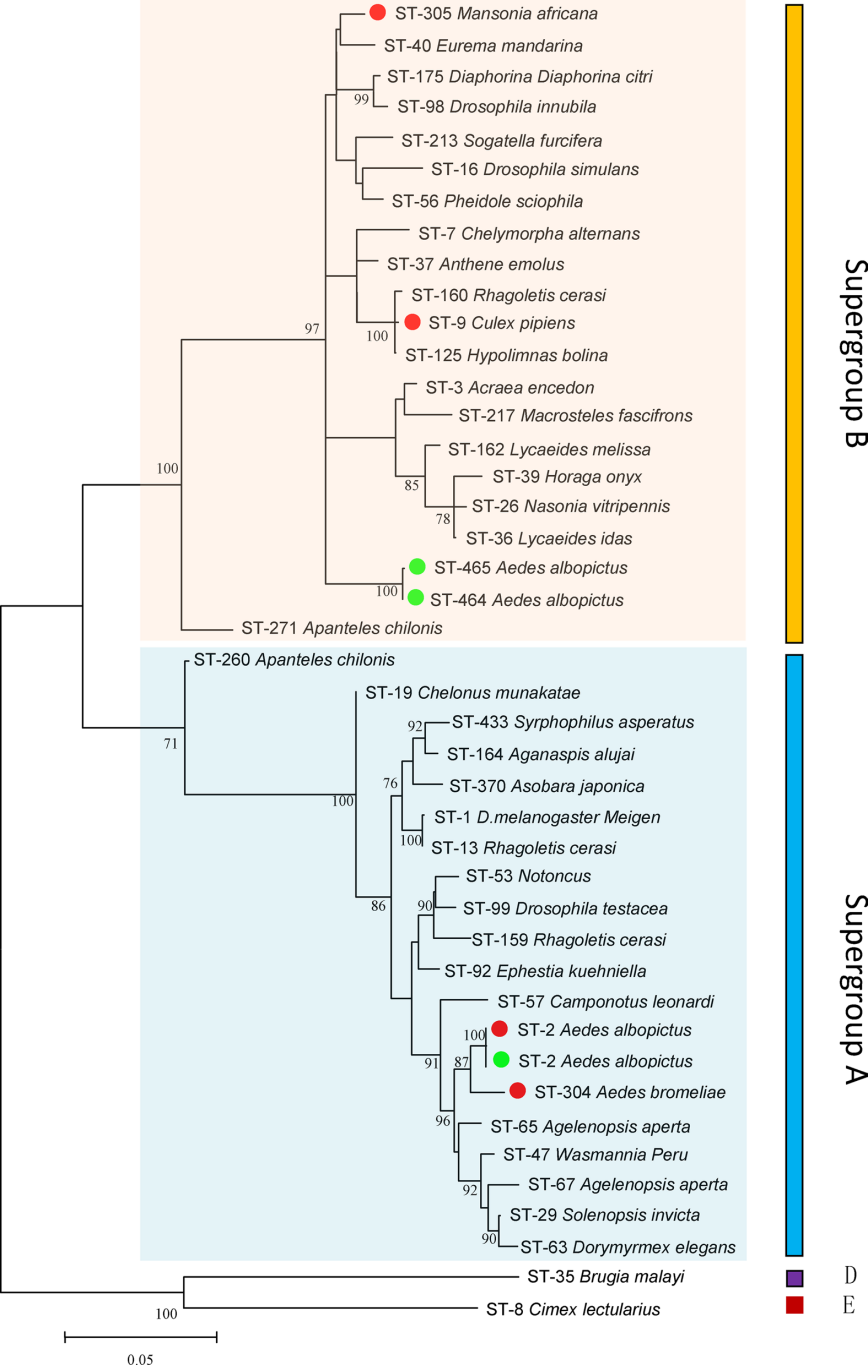
MLST-based maximum likelihood tree for *Wolbachia* from different hosts. Red dots indicate reported *Wolbachia* strains of mosquitoes; green dots indicate *Wolbachia* strains of *Ae. albopictus* sampled in the present study

**Fig. 6:**
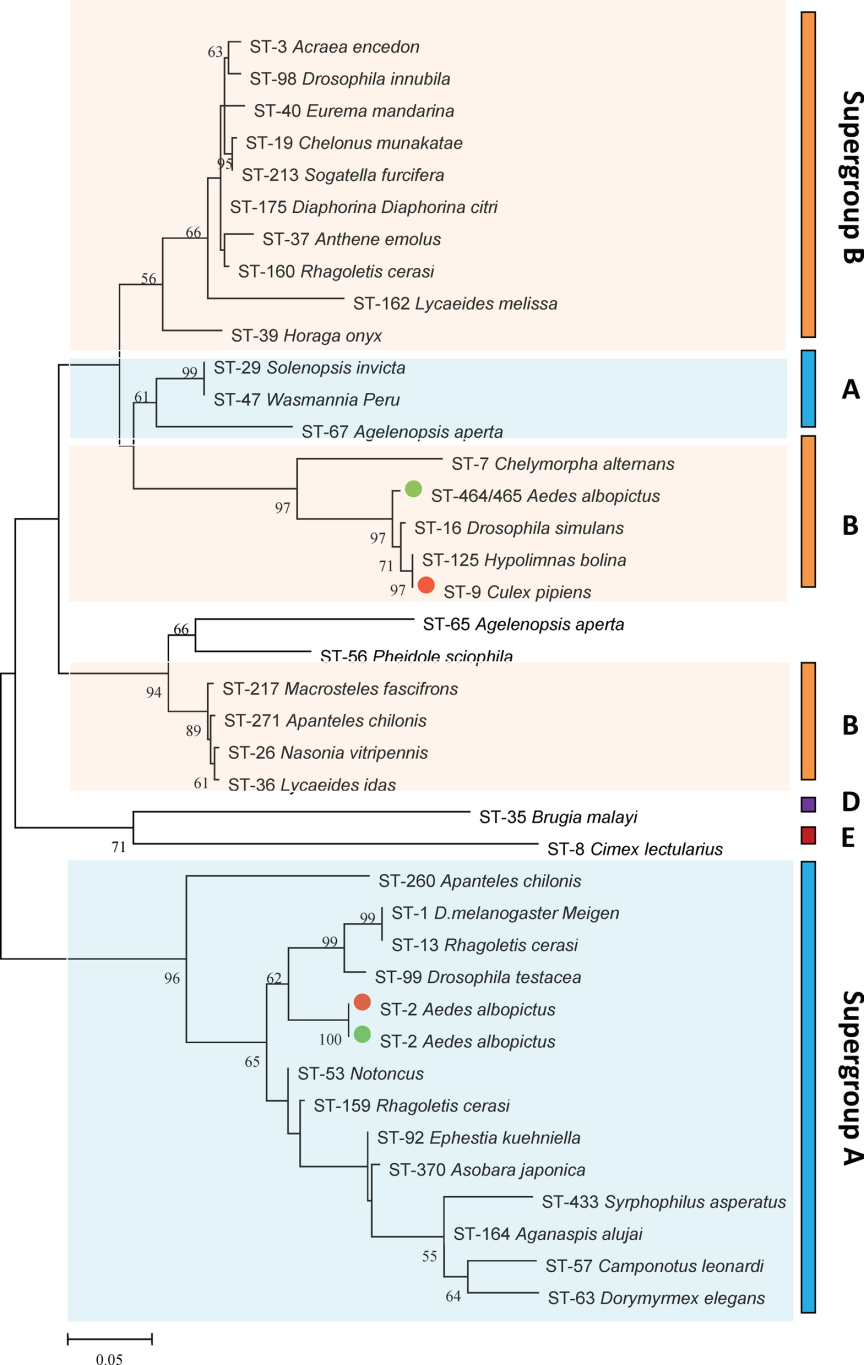
*wsp*-based maximum likelihood tree for *Wolbachia* from different hosts. Red dots indicate reported *Wolbachia* strains of mosquitoes; green dots indicate *Wolbachia* strains of *Ae. albopictus* sampled in the present study

Phylogenetic analysis based on the concatenated sequences of all MLST loci showed that ST-2 was *w*AlbA, but no closely-related STs were identified for *w*AlbB. We submitted our sequences to the MLST database, and received new ST codes (ST-464, ST-465, designated for *w*AlbB1, *w*AlbB2 respectively). *w*AlbB1 and *w*AlbB2 only differed by a single base pair: *gatB*16^A^ and *gatB*16^G^, respectively. The five MLST genes of *w*AlbA shared the same alleles as ST-2, as previously demonstrated [19]; however, three of the five MLST genes (*fbpA*, *gatB* and *hcpA*) of *w*AlbB1 and two of the five genes (*fbpA* and *hcpA*) of *w*AlbB2 shared alleles with other STs. In total, 40 known *Wolbachia* STs in the MLST database (http://pubmlst.org/Wolbachia/) were used as a dataset to infer the phylogeny of *Wolbachia* infecting field-collected *Ae. albopictus*. The MLST-based ML tree (Fig. 5) separated the isolates into three major clusters: supergroup A, supergroup B, and supergroup D + supergroup F. For the *wsp*-based ML tree, the isolates were separated into supergroup A, supergroup D, supergroup F and a mixture of supergroup A and supergroup B branches. According to these data, it was safe to classify ST-464 and ST-465 as strains of supergroup B. In the *wsp*-based ML tree (Fig. 6), *w*AlbA and *w*AlbB formed a cluster with strains from other mosquito species (*Culex quinquefasciatus* and *Culex gelidus*). Similarly, in the MLST-based tree, *w*AlbA (ST-2) formed a clade with ST-304 whose host is *Aedes bromeliae*. In supergroup B, *w*AlbB (ST-464 and ST-465) did not form a clade with ST-305 and ST-9, whose hosts were *Mansonia africana* and *Culex pipiens*, respectively (Figs. 5, 6).

### *w*AlbA and *w*AlbB *Wolbachia* strain quantitation

The relative densities of the *w*AlbA and *w*AlbB strains were estimated for individual females sampled from regions with local dengue cases, with only imported dengue cases, and without dengue cases. The data were normalized using the host *rps6* gene, which also allowed the densities of the two *Wolbachia* strains to be compared between different adult sizes.

**Fig. 7:**
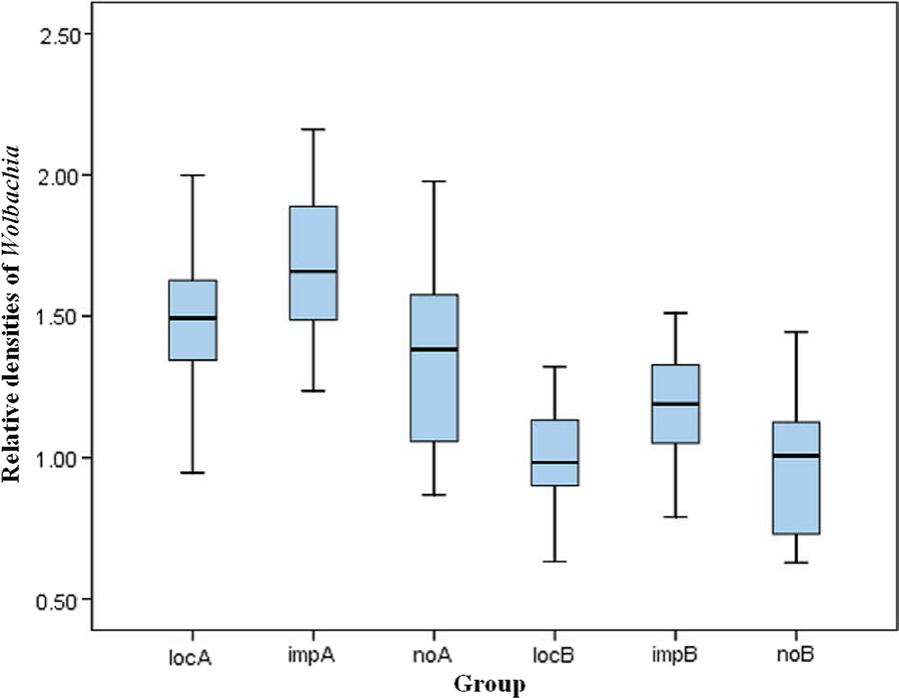
Relative *Wolbachia* densities in *Ae. albopictus* collected in different regions in China. *Abbreviations*: loc-A, relative densities of *w*AlbA in the regions with local dengue cases; imp-A, relative densities of *w*AlbA in the regions with import dengue cases; no-A, relative densities of *w*AlbA in the regions without dengue cases; loc-B, relative densities of *w*AlbB in the regions with local dengue cases; imp-B, relative densities of *w*AlbB in the regions with import dengue cases; no-B, relative densities of *w*AlbB in the regions without dengue cases

Figure 7 shows a higher density of the *w*AlbB strain relative to *w*AlbA, and this difference was significant in three different regions: locA *vs* locB (ANOVA, *F*_(1, 54)_ = 67.143, *P* < 0.0001), impA *vs* impB (ANOVA, *F*_(1, 54)_ = 38.955, *P* < 0.0001), and noA *vs* noB (ANOVA, *F*_(1,54)_ = 12.650, *P* = 0.001). Moreover, both *w*AlbA and *w*AlbB strains showed significantly lower densities in regions with only imported dengue cases than in the other two regions [*w*AlbA (ANOVA, *F*_(2, 81)_ = 10.203, *P* < 0.0001) and *w*AlbB (ANOVA, *F*_(2, 81)_ = 7.468, *P* = 0.001)]. Neither locA *vs* impA, locA *vs* noA, locB *vs* impB, nor locB *vs* noB showed any significant difference, which may indicate a relationship between the fluctuation of *Wolbachia* density in field *Ae. albopictus* and the invasion of dengue virus.

## Discussion

*Wolbachia* is a bacterial endosymbiont that infects the reproductive tissues of arthropods, mainly insects. It is spread primarily *via* the ova cytoplasm and alters the reproductive success of its host, thus making it a suspected driver of development and speciation. The prevalence of *Wolbachia* in insects has been reported as ranging from 20% to 65% [32]. Our results showed a prevalence of 93.36% for *Wolbachia* in natural populations of *Ae. albopictus* in China, slightly lower than the 100% previously reported in Guangzhou (China), Orissa (India), Chachoengsao (Thailand) [18, 33, 35] and over 99% in Korea [34]. Furthermore, single infections with both *w*AlbA and *w*AlbB were detected in our study and the prevalence of *w*AlbB (10.10%) strains was higher than that of *w*AlbA strains (2.31%). To the best of our knowledge, this is the first report of single *w*AlbB infections in field-collected *Ae. albopictus* in Changsha, Chenzhou and Wuhan, China, and our findings were similar to those reported in Guangzhou [18]. These results thus support and validate the work of O’Neill et al. [35]. In the present study, *28S* rRNA was used to assess the quality of DNA extraction [30] and the *cox*1 gene of *Ae. albopictus* was sequenced to rule out samples that were not *Ae. albopictus*. In addition, to obtain an accurate estimate of the prevalence of *w*AlbA and *w*AlbB, qPCR was used to check negative samples and indicated an increased prevalence of 83.26% and 91.05% for supergroup A and B *Wolbachia* strains, respectively.

*Wolbachia* significantly and efficiently reduced the proportions of mosquitoes achieving infection and transmission potential across the different regions. *Wolbachia* density is sensitive to temperature variations [36]. A Chi-square test of *Wolbachia* prevalence among the five different climate regions in China revealed that geographical location and climate may have a significant effect on the prevalence of *Wolbachia* in natural populations of *Ae. albopictus*. As shown in Fig. 3, for both *w*AlbA and *w*AlbB, the prevalence of *Wolbachia* infection in the Mid-subtropical region was lower than in other climate regions; the difference between the North subtropical region and the Warm temperate zone was apparent in all three measures of prevalence. There was a clearly lower prevalence in Chenzhou (Fig. 2), which may be the reason why rates in the Mid-subtropical region were lower than in other regions. Aside from this, the rates of *Wolbachia* infection did not show any linear relationships, which may imply that there is no absolute correlation between climate region and *Wolbachia* infection.

MLST is an important source of sequence data for comparative genetics, providing a tool for exploring molecular evolutionary methods in intracellular bacteria [19]. Our results show that in both the MLST-based and *wsp*-based ML trees, *Wolbachia* isolates included in the analyses are placed in supergroups A and B (Fig. 5). However, in the *wsp*-based ML tree (Fig. 6), a mixed cluster of supergroups A and B was identified, with ST-19, ST29, ST47, ST65 and ST67 belonging to a supergroup associated with isolates from supergroup B. This suggests that MLST-based genotyping is perhaps more accurate than the *wsp*-based method. Our results may, however, be explained by the fact that the sharing of *wsp* sequences between A and B strain supergroups indicates a strong genetic cohesiveness of *Wolbachia* strains [37]. Moreover, for supergroup B in the *wsp*-based ML tree, *Wolbachia* of *Ae. albopictus* did not show an exact match with previously identified STs. Furthermore, we identified the new ST-464 strain *w*AblB1 and the new ST-465 strain *w*AblB2. ST-464 was found in all locations, but ST-465 strains were only found in single infected mosquitoes from Changsha and Chenzhou and co-infected mosquitoes from Wuhan and Nanchang. This may reflect the various states of *Wolbachia* infection in these locations.

The density of the endosymbiont *Wolbachia* plays an important role in crossing sterility, which is known as a cytoplasmic incompatibility and limits the degree of parental spread. *Aedes albopictus* mosquitoes can be superinfected with the *Wolbachia* strains *w*AlbA and *w*AlbB [38]. In our study, the *w*AlbB strain was found at a higher density than *w*AlbA in *Ae. albopictus*, which is consistent with the results of two previous studies [38, 39]. To our knowledge, this study is the first to assess relative *Wolbachia* densities in *Ae. albopictus* mosquitoes from different natural populations, which were sampled from regions with different dengue fever load. The relative density of *Wolbachia* (*w*AlbA and *w*AlbB) in mosquitoes from regions with only imported dengue cases was lower than that in mosquitoes from regions with local dengue cases and without dengue cases. The decrease of *Wolbachia* density could lead to the loss of protection by the host immune system [40]. We hypothesize that the imported dengue cases caused a lowering of *Wolbachia* densities in natural mosquito populations and that densities of virus in these mosquitoes will increase. Sometime later, densities of virus and *Wolbachia* would come to a balance in the natural mosquito populations and thereafter could transmit virus smoothly, resulting in local dengue case emerging. This hypothesis has yet to be substantiated by other reports, but our results may reflect the alarm reaction of natural mosquito populations in response to invasion of dengue virus, which is embodied in the fluctuation of *Wolbachia* densities. Furthermore, the low prevalence in Chenzhou, which also has imported dengue cases, may be explained if our hypothesis were correct [41, 42]. Further research is needed to explore the relationship between *Wolbachia* densities in natural *Ae. albopictus* mosquitoes and the invasion of dengue virus.

In this study, we obtained adult mosquitoes at a variety of ages from different parts of China. Because adults had only recently emerged (1 or 2 days), these may have had *Wolbachia* densities that were too low to be detected. Our subsequent studies will be based on field-collected larvae, which will be brought back to the laboratory and used for further research after emergence.

## Conclusions

This study demonstrated that the natural prevalence of *Wolbachia* infections in China was much lower than the prevalence in other countries or regions. The prevalence of *Wolbachia* was significantly different among five different climatic regions. The phylogenetic relationships of *Wolbachia* in field-collected *Ae. albopictus* were estimated based on MLST and *wsp* analyses, and showed that these strains were rather stable. However, *w*AlbB (464/465) and *Wolbachia* strains did not form a clade with *Wolbachia* strains from other mosquitoes. Moreover, the lower densities of *Wolbachia* in regions with only imported dengue cases suggested a relationship between the fluctuation of *Wolbachia* density in natural *Ae. albopictus* populations and the invasion of dengue virus.

## Data Availability

The data supporting the conclusions of this article are included within the article. Representative nucleotide sequences generated in this study were deposited in the GenBank database under the accession numbers KU738304-KU738385 and KU738386-KU738431. The sequences of *gatB*, *coxA*, *hcpA*, *ftsZ*, *fbpA* and *wsp* were deposited in the MLST database and the allele numbers are 247/242, 229, 166, 210, 27 respectively.

## Abbreviations

MLST: multilocus sequence typing
ST: sequence type
PCR: polymerase chain reaction
ddH_2_O: double distilled water
WSP: *Wolbachia* surface protein gene
HVR: hypoxic ventilatory response
