# Correction to: Identification and molecular characterization of *Wolbachia* strains in natural populations of *Aedes albopictus* in China

**DOI:** 10.1186/s13071-020-3917-6

**Published:** 2020-03-12

**Authors:** Yaping Hu, Zhiyong Xi, Xiaobo Liu, Jun Wang, Yuhong Guo, Dongsheng Ren, Haixia Wu, Xiaohua Wang, Bin Chen, Qiyong Liu

**Affiliations:** 1 State Key Laboratory of Infectious Disease Prevention and Control, Collaborative Innovation Center for Diagnosis and Treatment of Infectious Diseases, WHO Collaborating Centre for Vector Surveillance and Management, National Institute for Communicable Disease Control and Prevention, Chinese Center for Disease Control and Prevention, Beijing, 102206 People’s Republic of China; 2 Nanjing Institute of Environmental Sciences, Ministry of Ecology and Environment of the People’s Republic of China, Nanjing, China; 3 Institute of Entomology and Molecular Biology, College of Life Sciences, Chongqing Normal University, Chongqing, China; 4 Key Laboratory of Tropical Disease Control of the Ministry of Education, Sun Yat-sen University-Michigan State University Joint Center of Vector Control for Tropical Diseases, Zhongshan School of Medicine, Sun Yat-sen University, Guangzhou, China; 5 Haikou Center for Disease Control and Prevention, Haikou, China

## Correction to: Parasites Vectors (2020) 13:28 10.1186/s13071-020-3899-4

Following publication of the original article [1], the corresponding author flagged that the article had published with two errors.

The first error concerns affiliation ‘1’, which was incomplete; please find the complete reference in this correction.

While the second is that an incorrect version of Fig. 7 had been used.

For reference, please find (the correct version of) Fig. 7 in this article.

**Fig. 7:**
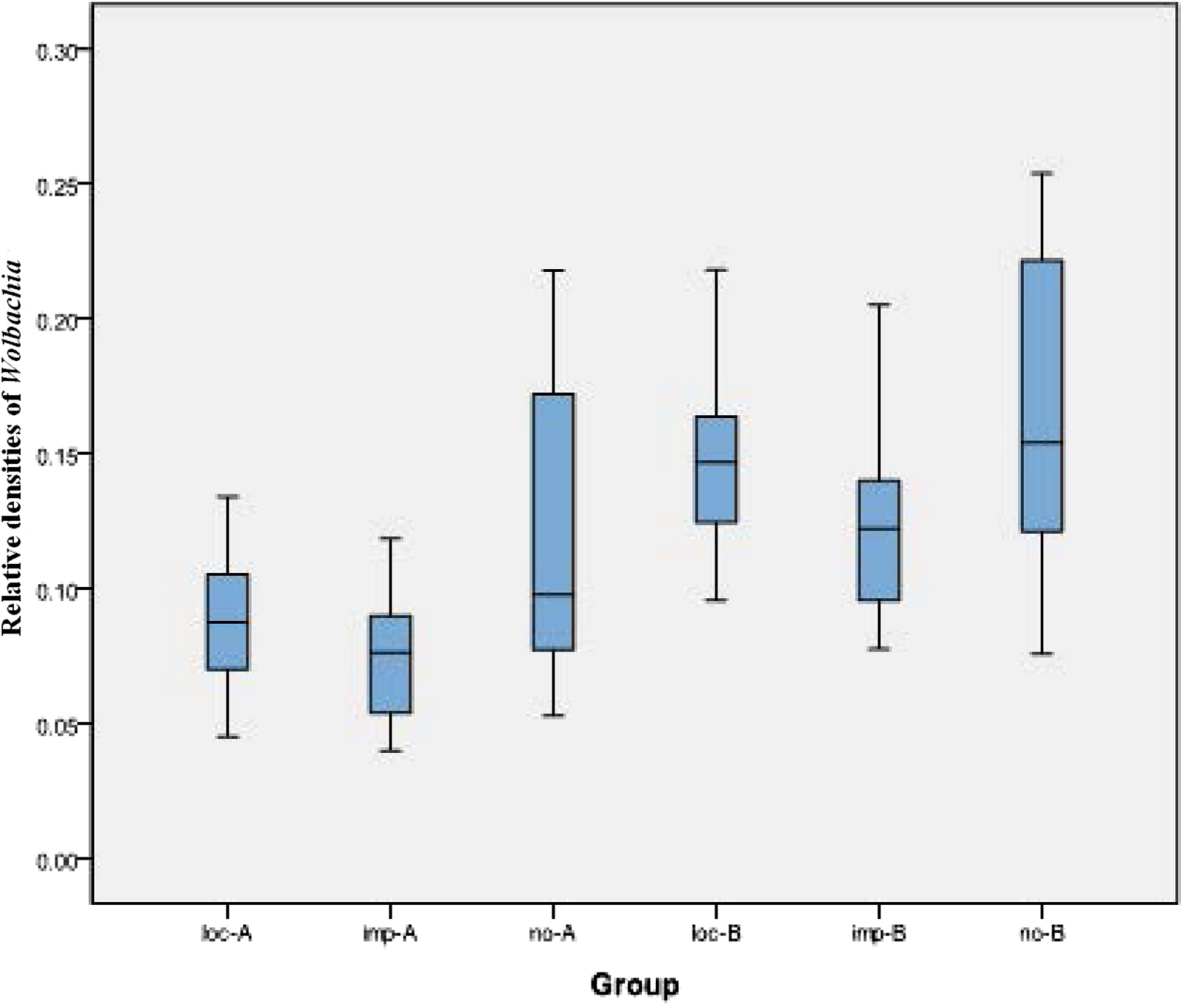
Relative *Wolbachia* densities in *Ae. albopictus* collected in different regions in China. *Abbreviations*: loc-A, relative densities of *w*AlbA in the regions with local dengue cases; imp-A, relative densities of *w*AlbA in the regions with import dengue cases; no-A, relative densities of *w*AlbA in the regions without dengue cases; loc-B, relative densities of *w*AlbB in the regions with local dengue cases; imp-B, relative densities of *w*AlbB in the regions with import dengue cases; no-B, relative densities of *w*AlbB in the regions without dengue cases

The authors apologize for any inconvenience caused.
